# A System for Converting and Recovering Texts Managed as Structured Information

**DOI:** 10.1038/s41598-022-26304-w

**Published:** 2022-12-23

**Authors:** Edgardo Samuel Barraza Verdesoto, Marlly Yaneth Rojas Ortiz, Richard de Jesus Gil Herrera

**Affiliations:** 1 Universidad Americana de Europa (UNADE), Cancún, México; 2 Universidad Internacional de la Rioja, Logroño, Spain; 3 Research Department, Tecnológica Autónoma de Bogotá (FABA), Bogotá, Colombia; 4 Universidad de Santander (UDES), Bogotá, Colombia

**Keywords:** Computer science, Information technology, Software

## Abstract

This paper introduces a system that incorporates several strategies based on scientific models of how the brain records and recovers memories. Methodologically, an incremental prototyping approach has been applied to develop a satisfactory architecture that can be adapted to any language. A special case is studied and tested regarding the Spanish language. The applications of this proposal are vast because, in general, information such as text way, reports, emails, and web content, among others, is considered unstructured and, hence, the repositories based on SQL databases usually do not handle this kind of data correctly and efficiently. The conversion of unstructured textual information to structured one can be useful in contexts such as Natural Language Generation, Data Mining, and dynamic generation of theories, among others.

## Introduction

Written communication is a type of information that has a basic structure well defined in each language which is useful in information processing^1^. Additionally, some other types of communication can be totally or partially converted into text whereupon the final processing is carried out^2,3^.

Several applications have been developed in information processing following principles or attributes of the text, for instance, to build ontologies or micro-theories^4–7^ which are convenient for automatic decision-making tasks^8^. Furthermore, In process automation, the speed at which the information is produced reduces human performance and delays the decision-making processes, this has generated the urgent need to delegate some decisions to machines and create applications for resolving these problems^9–11^.

The information retrieval and the generation of natural language promote the generation of sentences/phrases with meaning from a large amount of data generated by interactivity, shared repositories, and homogeneous or heterogeneous data sources, one of these types of application is presented in^12^ where is exposed a framework able to generate three classes of question and answers from corpora: *fill in the gaps*, *multiple choice*, and *shuffled sentences*. The framework aims to create a pedagogical tool able to automatically generate tests in the context of a topic, the parser divides texts according to the processed language and prepares the type of question selected. These approaches that allow recording and recovering of fragments or whole texts from a repository, conceiving and improving strategies applied in recovering unstructured information are very important in the current Computer Science.

This article introduces an architecture that allows building applications capable of dissociating texts/sentences in subsets of cores with properties and simple operations such as those that the algebraic groups incorporate. These operations are preferable because they have properties that promote a straightforward and reliable manner to retrieve a whole text or part of it by keeping the structure of the language. Additionally, relationships between subsets are incorporated because they play an essential role in maintaining the meaning of the recovered text/sentences.

The main objective of this manuscript is to show the design of a system with the capability of processing sentences (part of the text), storing them in databases, and finally, recovering them while keeping the original text’s basic meaning. To reach that, it is reviewing some previous concepts and experiences about linguistic computational to support the architectural design; it is described and justified how the algebraic groups help in the organization of the components of sentences for storage and recovering them while keeping the meaning and structure; Also, it is treated how to design an architecture for a processing system the objects/data as structured information (into structured databases), and finally, it is shown the functionality of a system for some illustrative instances and test cases for the Spanish language.

This paper is organized as follows. Firstly, a theoretical framework under which the proposal is based will be explored. Secondly, a general architecture will be proposed that incorporates each one of the elements exposed in the theoretical framework. Thirdly, an approach, based on the architecture presented, applied to the Spanish Language will be analyzed. Finally, the findings and future works will be exhibited.

## Conceptual framework

### Object-action dissociation/integration

These studies and approaches suggest that the information held in the brain is a set of clusters (cores) that could be affected by the ambiguity and the context in both, the dissociation and integration. Likewise, according to some theories, the brain saved our memories in two ways: semantically and the episodic way^13,14^, this latter manner is very important to explain the development of the strategy followed in this paper.

Historically, the dissociation of the information by the human brain was observed when comparing *Broca’s aphasic agrammatical patients*, whose speech involves the use of very few verbs in contrast with other *anomic patients* that had great difficulty finding concrete nouns^15^. Initially, the major difficulty with verbs for Broca’s patients was interpreted based on the highest syntactical complexity of verbs compared to nouns^16–18^. However, the idea that verbs are, in general, harder to produce has been undermined in other studies where it is indicated that patients with anomic difficulties produce verbs more easily than nouns^19,20^. From a *neurophysiological* point of view, there are different opinions and theoretical proposals^21^, of which three hypotheses have been put forward regarding verb-noun storage issues within neural networks: *partial separation of verbs and nouns*^22,23^, *word separation based on morphosyntax*^24^, and *separation between actions and objects*^25,26^. Psycholinguistics also agree that exist, in the brain, the distinction between various grammatical categories, particularly between verbs and nouns, and propose three possible starting points or context to access the information: *availability of information related to the grammatical class*, *a required grammatical knowledge*, and *the independence between the definition of grammatical class and the semantic differences*^27–30^. A considerable number of studies have dealt with aspects associated with the dissociation of the information within the human mind and the conclusions are similar^31^, there are a dissociation between verbs(actions) and nouns (objects).

The counterpart of the dissociation process is the integration process. According to^32^, grammatical information is relevant to understanding and producing sentences, but a plausible conclusion suggests that the grammatical class information is not a lexical property that can be retrieved automatically; instead, this property is likely to play an important role in the context of a sentence. Fundamentally, the role of the grammatical class in sentence processing is modulated by the linguistic differences regarding the way as words of certain grammatical classes are used within sentences. In all languages, verbs commonly require higher processing than nouns at various levels, firstly, because the processing of verbs is about events and could exist many elements that will have to be integrated. Secondly, the verb syntax also demands more processing because verbs should be connected to other words to convey their meaning. Lastly, nouns are linked to objects, but they might refer to events too, and it is necessary its disambiguation. In conclusion, the effects of the grammatical class in the retrieval and representation of simple words are more productive when the context is present^33–35^.

In addition, Neuroscience states that there are two kind of memory for storing and remembering facts and events consciously; such events are stored in the *episodic memory* such as a storyteller whereas the *semantic memory* records the same event as part of our overall knowledge (dictionary). In specific, the *episodic memory* is intended as a repository in our brain where is recorded an event similar to a text well-written^13,14^.

In summary, there are two processes well conceived in our brain, dissociation and integration of an event. These tasks are the fundamentals of the proposal in this paper.

### From sentences to clusters of words

**Figure 1:**
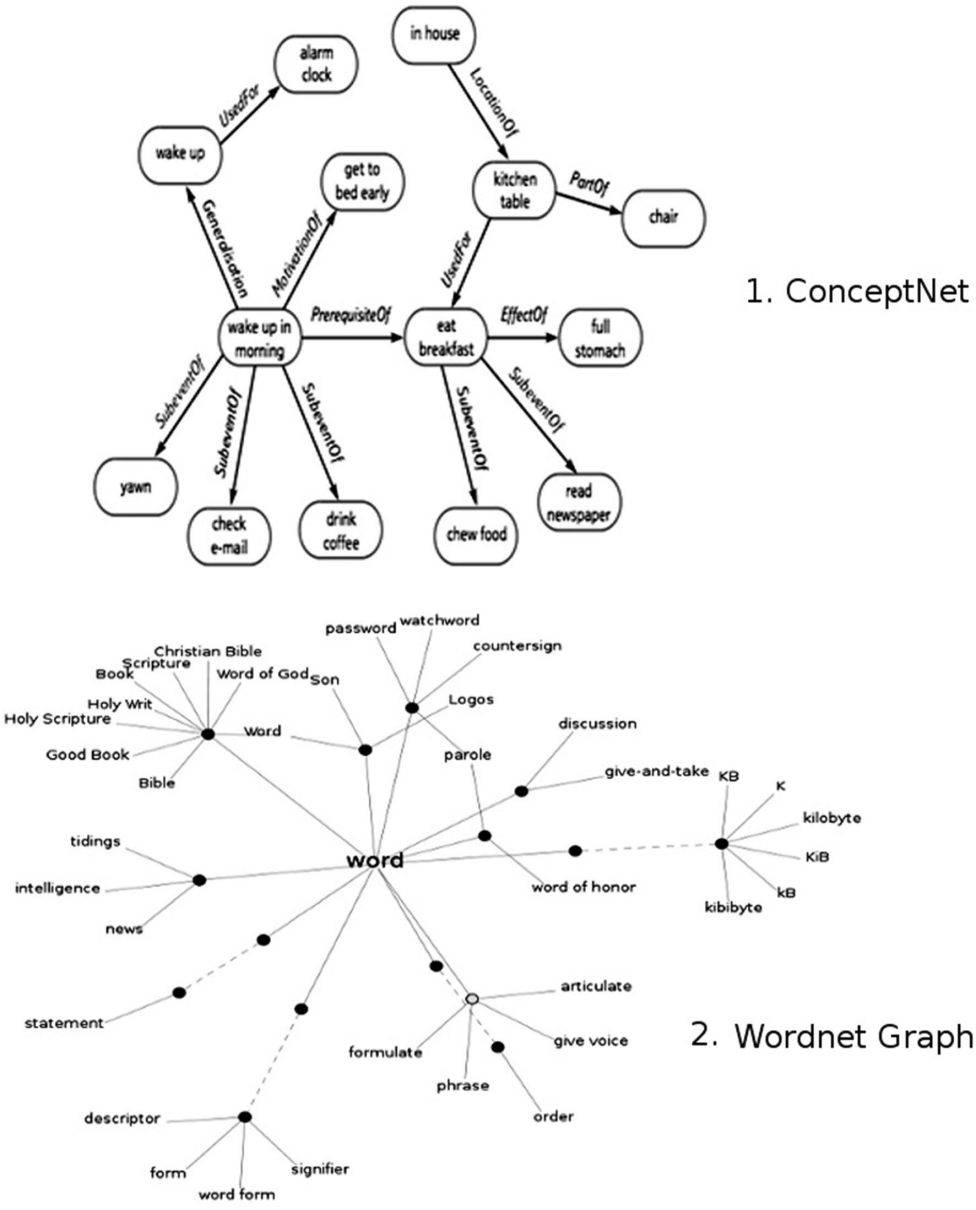
Some word classifications techniques. Source: Extracted and adapted from^37,42^.

The word classification has been a normal practice in linguistics, computer sciences, and education, among others (see Fig. 1); this practice normally has different targets and results. Furthermore, as an instance, ConceptNet is a project based on the sense common concept that was conceived as a semantic network containing lots of things that the computers should know about the world^36–38^. Another example is the WordNet project which resembles a thesaurus in that words are grouped based on their meanings, the result is a network that can be browseable easily^39–42^.

A text is more complex than simple words, it is a texture that relates, firstly, words to create sentences, secondly paragraphs, and ignoring other structures, finally several paragraphs directly or indirectly (e.g. using the anaphor) linked between one another result in a text. Each language has rules to build sentences and paragraphs. According to^43^, there are various ways to classify and describe the languages, but a very common is the order of each one of their main components (**Subject**, **Object**, and **Verb**) in the sentence:

- *Subject-Object-Verb* (**SOV**). This is the most frequent type of word order in spoken languages.
- *Subject-Verb-Object* (**SVO**). It is a relevant type of word order because of its speakers worldwide.
- *Verb-Subject-Object* (**VSO**): It represents a relatively small set of languages.
- *Verb-Object-Subject* (**VOS**): Very few languages use this kind of order.

Some approaches use such classifications to divide sentences, expressions, paragraphs, and texts, and, ultimately, to generate categories that are used in specific applications^44,45^. Additionally, other applications use these characteristics in a reverse way, for instance, to build sentences and paragraphs, or concatenate textual expressions from the same or different sources for generating new expressions; this is being applied in Human Machine Interfaces (**HMI**) development^46^. On the other hand, a text not only has nouns and verbs, else other types of words with different purposes, e.g., emphasizing words, which to join small sentences to produce effects like generalization or itemization, etc. These words play an important role to decide how the relations between words, sentences, and paragraphs are. They can be linked to verbs or nouns, e.g. the determinants which comply with the function of generalization or quantification of nouns^47^.

In^48^ was analyzed the preliminary results focused on the dissociation of sentences in clusters. The sentences studied were in the Spanish Language. Section 2 of this reference exposes why it is necessary to migrate from String-set dependence to another algebraic structure for modeling a sentence, and why this algebraic structure must be an Abelian group, it also supplied the proof. In summary, the dissociation between verbs and nouns, mainly, is a convenient strategy to generate new sentences, also, it is important to create an adequate environment for it.

### Algebraic environment

Modern Algebra is a discipline that deals with the properties of the sets and their elements, and the operations that can be executed within them. Modern algebra classifies the sets as **semigroups**, **monoids**, **groups**, **rings**, and **fields**; all of these are named algebraic structures. These classifications depend on the number and type of properties that the operation fulfills.

If the elements of sentences are treated like components of an algebraic set, then such components could be used to build phrases and new sentences easily by applying an operation that complies with certain properties. This section shows that converting the conventional algebraic structure of the set of strings (sentences) to a structure more adequate allows for reaching this purpose.

A class very important for this approach is the *groups*, specifically, the **Abelian groups**^49^ these last ones have significant properties that guarantee that by operating elements of a dissociated sentence, the original sentence can be rebuilt; A key property is to be commutative because it allows that the result of an operation among elements will be the same, although the operands change their place in the operation.

A sentence could be treated as an ordered set of strings which implies an algebraic structure very simple, but this structure does not is adequate because each string in the sentence complies with a function depending on its position in it, if the sentence is dissociated and then it is reassembled, this last process must guarantee that the product is at least coherent with the structure of the language.

A sentence could be treated as an ordered set of strings which implies an algebraic structure very simple, it is ordered because each string in the sentence complies with a function depending on its position in it, if the sentence is dissociated into strings and later is required its reassembling, this last process must guarantee that the final sentence keeps the structure of the language and its meaning. These conditions comply if the set generated in the dissociation has associated an operation with certain properties which will be shown in this section.

Supposing the following sentence in the Spanish language: *”Fred quiere ir a Hong Kong y visitar sitios turísticos”* (*English meaning: ”Fred wants to go to Hong Kong and visit tourist places”*), and it is dissociated in strings with a word each one. One scenario for creating Natural Language from this dissociation will be to use the **conventional algebraic structure of strings** which is composed of the set of strings, and an operator able to join the strings and generate others (closure property). In this algebraic structure the closure property functions as follows:

$$\begin{aligned} \hbox {String-set} = \lbrace ``\hbox {Fred}'', ``\hbox {Hong Kong}'', ``\hbox {quiere}'', ``\hbox {ir}'', ``\hbox {a}'', ''\hbox {y}'', ''\hbox {visitar}'', ''\hbox {sitios}'', \hbox {turisticos}{\rbrace }\\ \hbox {A new string} = ``\hbox {Fred}'' + ``\hbox {quiere}'' + ``\hbox {ir}'' + ``\hbox {a}'' + ``\hbox {Hong Kong}''\\ = ``\hbox {Fred quiere ir a Hong Kong}'' \end{aligned}$$

But the closure property is not enough, because the generation of a new string in natural language must ensure structure and meaning, and this is not completely possible in this set with this operation, for example:

$$\begin{aligned} \hbox {A new string} = ``\hbox {ir}'' + ``\hbox {quiere}'' + ``\hbox {a}'' + ``\hbox {Fred}'' + ``\hbox {Hong Kong}''\\ = ``\hbox {ir quiere a Fred Hong Kong}'' \hbox {(no meaning)} \end{aligned}$$

**Figure 2:**
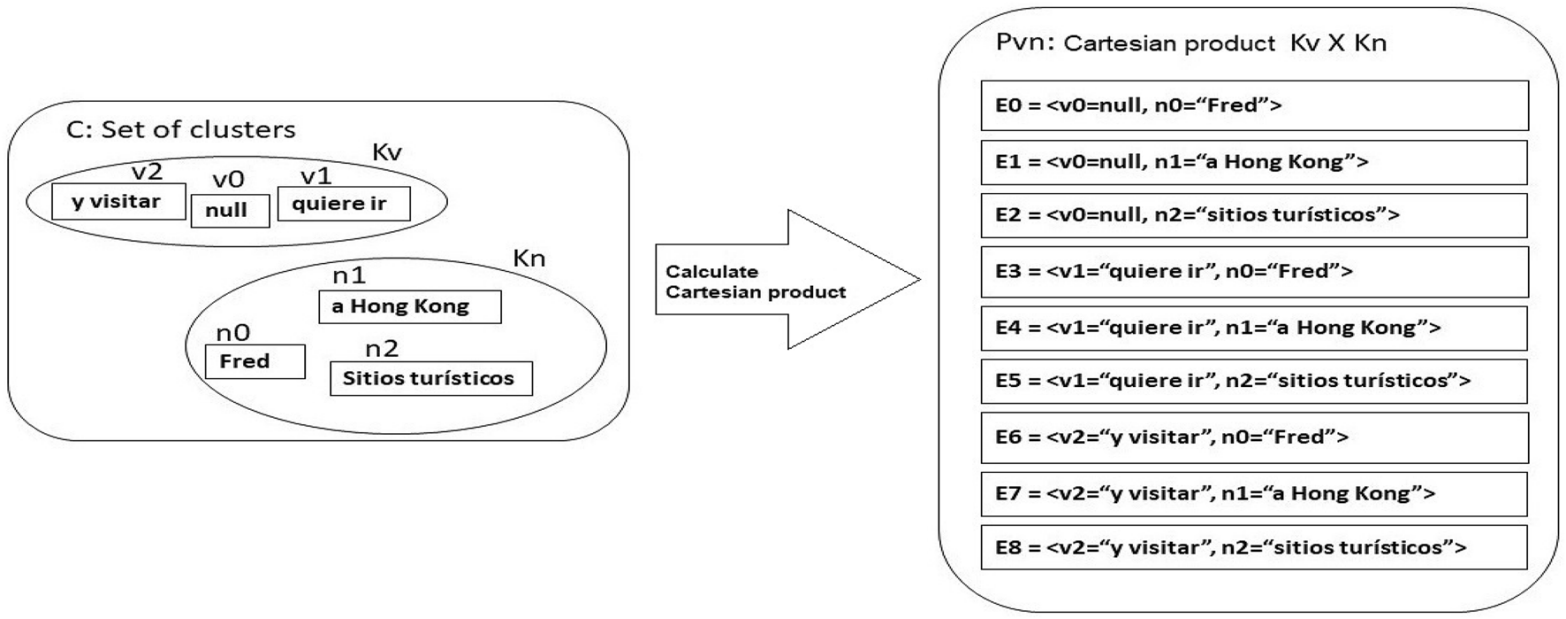
Dissociation of a sentence. Source: Own elaboration.

A possible solution is to divide the sentence into adequate strings forming ordered sets of clusters. The sets generated by this process will be named **Kn** (noun-cores/noun-clusters) and **Kv**(verb-cores/verb-clusters)), but the set used to generate phrases would be the Cartesian product of these sets ($Kv \times Kn = P_{vn}$). In this strategy, the verb-core must contain the null string because the SVO languages (Spanish and English, among others) allow generating phrases without verbs. Figure 2 shows a dissociation following the heuristics in^48^:

The operation should destroy the operand pairs and apply the operation concatenation or plus (+). This method can generate several sentences, but any of them without meaning, or, at least in the context of the original sentence:

$$\begin{aligned} \hbox {E}0 + \hbox {E}3= & {} \hbox {null}+``\hbox {Fred}''+''\hbox {quiere ir a}''+``\hbox {Hong Kong}''\\= & {} ``\hbox {Fred quiere ir a Hong Kong}'' \hbox {(correct)}\\ \hbox {E}2 + \hbox {E}4= & {} \hbox {null}+``\hbox {sitios turisticos}''+''\hbox {quiere ir}''+''\hbox {a Hong Kong}''\\= & {} ``\hbox {sitios turisticos quiere ir a Hong Kong}'' \hbox {(no meaning)} \end{aligned}$$

So far, this strategy revolves around the *closure property* and other properties such as *associative*, and the *neutral element*; but this is not enough to guarantee structure and meaning, at least compared to the source text. To improve this proposal is necessary to include more properties to the set along with the operation, this is only possible by exploring other possible set types that can build up an algebraic structure more useful, and thus, it is decisive to map $P_{vn}$ to another set that will be named $O_{vn}$. Table 1 shows the new set and its components.

**Table 1:** Set $O_{vn}$ and description of its components. Source: Own elaboration.

Components	Description
$\llbracket [v_0:v_1:\cdots :v_k],$ $[n_0:n_1:\cdots :n_k]\rrbracket$ or $\llbracket X\rrbracket$	**Components:** Each component is a vector of vectors. First-internal-vector only contains verbal clusters (v-elements). Second-internal-vector only nominal clusters (n-elements). $\llbracket X\rrbracket$ generalizes the internal vectors.
$\lambda _k$	$\varvec{\lambda }-{element:}$ Represents a null string. The subscript is the position in the internal vector.
$\llbracket [\lambda _{0/\infty }],[\lambda _{0/\infty }\rrbracket$	$\varvec{\Gamma }-{element}$ A component with only $\lambda -elements$ in all internal vectors.

**Mapping** $F_{vn}: P_{vn} \rightarrow O_{vn}$: Let us define $F_{vn}$ as:

- Pairs $<v_i, n_j>$ belong $P_{vn}$ with different index ($i \ne j$) will be mapped to $\Gamma$ in $O_{vn}$.
- All pairs mapped must contain at least a *n-element*, then, pairs such as $<v_i, \lambda>$ will be mapped to $\Gamma$ in $O_{vn}$.
- Additionally, if couples such as $<v_i, n_i>$ exist in $P_{vn}$, then, elements such as $<\lambda , n_i>$ will be mapped to $\Gamma$ in $O_{vn}$.

**Figure 3:**
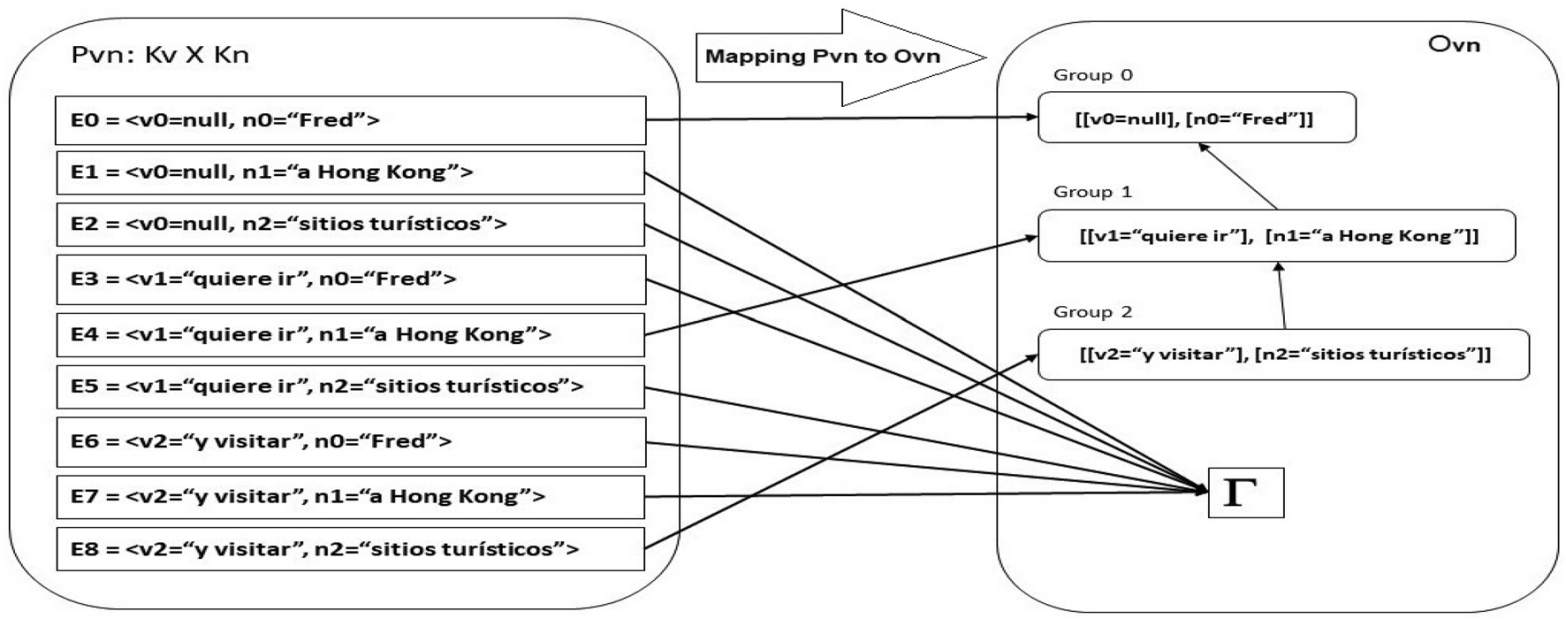
Dissociation of a Spanish sentence. Mapping from $P_{vn}$ to $O_{vn}$. Source: Own elaboration.

Figure 3 show the mapping made from $P_{vn}$ to $O_{vn}$ for the example.

In $O_{vn}$ the operation used, also, change, and it is defined as follows:

- **Dual**. It is *Dual* because of whether two components are operated, then the operation takes place independently in each internal vector. This property allows to separate completely verbs and nouns.
- **Positional**. It is *Positional* because the operation is carried out by two elements with the same subscript. This property allows to implement commutativity.

For example:

$$\begin{aligned}{} & {} {{\llbracket [\lambda _0:v_1:\lambda _2] [\lambda _0:n_1:\lambda _2]\rrbracket }} + {{\llbracket [\lambda _0:\lambda _1:v_2] [\lambda _0:\lambda _1:n_2]\rrbracket }} \\= & {} {{\llbracket [\lambda _0+\lambda _0:v_1+\lambda _1:\lambda _2+v_2] [\lambda _0+\lambda _0:n_1+\lambda _1:\lambda _2+n_2]\rrbracket }} \\= & {} {{\llbracket [\lambda _0:v_1:v_2] [\lambda _0:n_1:n_2]\rrbracket }} \\= & {} v_1+n_1+v_2+n_2 \end{aligned}$$

This operation is the same that:

$$\begin{aligned} ?  {} & {}{{\llbracket [\lambda _0:''quiere\quad ir'':\lambda _2] [\lambda _0:''a\quad Hong\quad Kong'':\lambda _2]\rrbracket }} + {{\llbracket [\lambda _0:\lambda _1:''y\quad visitar''] [\lambda _0:\lambda _1:''sitios\quad turisticos'']\rrbracket }} ? \\  = ?& {} {{\llbracket [\lambda _0+\lambda _0:''quiere\quad ir''+\lambda _1:\lambda _2+''y\quad visitar''] [\lambda _0+\lambda _0:''a\quad Hong\quad Kong''+\lambda _1:\lambda _2+''sitios\quad turisticos''] \rrbracket }} ?  \\ =  ?  & {} {{\llbracket [\lambda _0:''quiere\quad ir'':''y\quad visitar''] [\lambda _0:''a\quad Hong\quad Kong'':''sitios\quad turisticos'']  \rrbracket }} ?\\ \end{aligned}$$

To create the sentence starting from this new core is applied a process that states that for each position in the vectors a verb is concatenated with the noun corresponding and the result will be added to the next result as follows:

$$\begin{aligned}= & {} ''(quiere\quad ir''+''a\quad Hong\quad Kong)'' \\= & {} ''quiere\quad ir''+''a\quad Hong\quad Kong''+''(y\quad visitar''+''sitios\quad turisticos)'' \\= & {} ''quiere\quad ir\quad a\quad Hong\quad Kong\quad y\quad visitar\quad sitios\quad tursticos'' \end{aligned}$$

It is too easy to deduct that this new operation in $O_{vn}$ is commutative, i.e., the result is the same, although the operands will change their position. This commutative structure is known as an **Abelian monoid structure** and, in^48^, and by including the **symmetrical element**, is converted to an Abelian group.

An algebraic structure as has been defined is very useful because reduces the complexity in the reconstruction of phrases because the operation is easy to implement and its behavior is similar to the add operation in the numbers by managing sentences as sets of discrete cores. In the section ”An approach” will be explained that a sentence can generate several Abelian groups, and each one can generate sentences separately.

## Methodology

**Figure 4:**
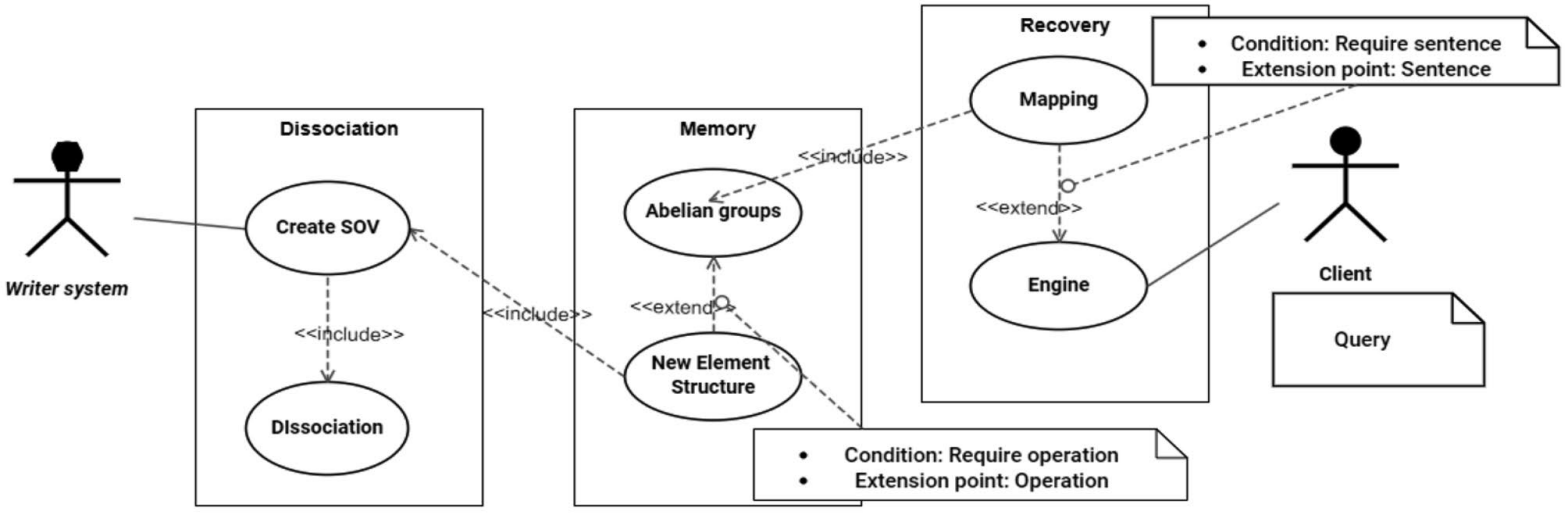
Use case for the architecture of the system. Source: Own elaboration.

This section proposes an architecture of a system, for dissociating and recovering texts and sentences, based on the concepts, theories, and regulations aforementioned. Figure 4 shows a scheme of the system based on use cases view^50^. The system would include three major sub-systems: **dissociation**, **memory** and **recovery**. The two first sub-systems will be activated serially and immediately after a reading takes place, and the latter process is executed when a query promotes the generation of sentences. Nevertheless, in terms of the information processing associated with each sub-system, they operate independently. The entire system is conceptualized as a framework that could be up-gradable and enriched with plug-in modules.

**Figure 5:**
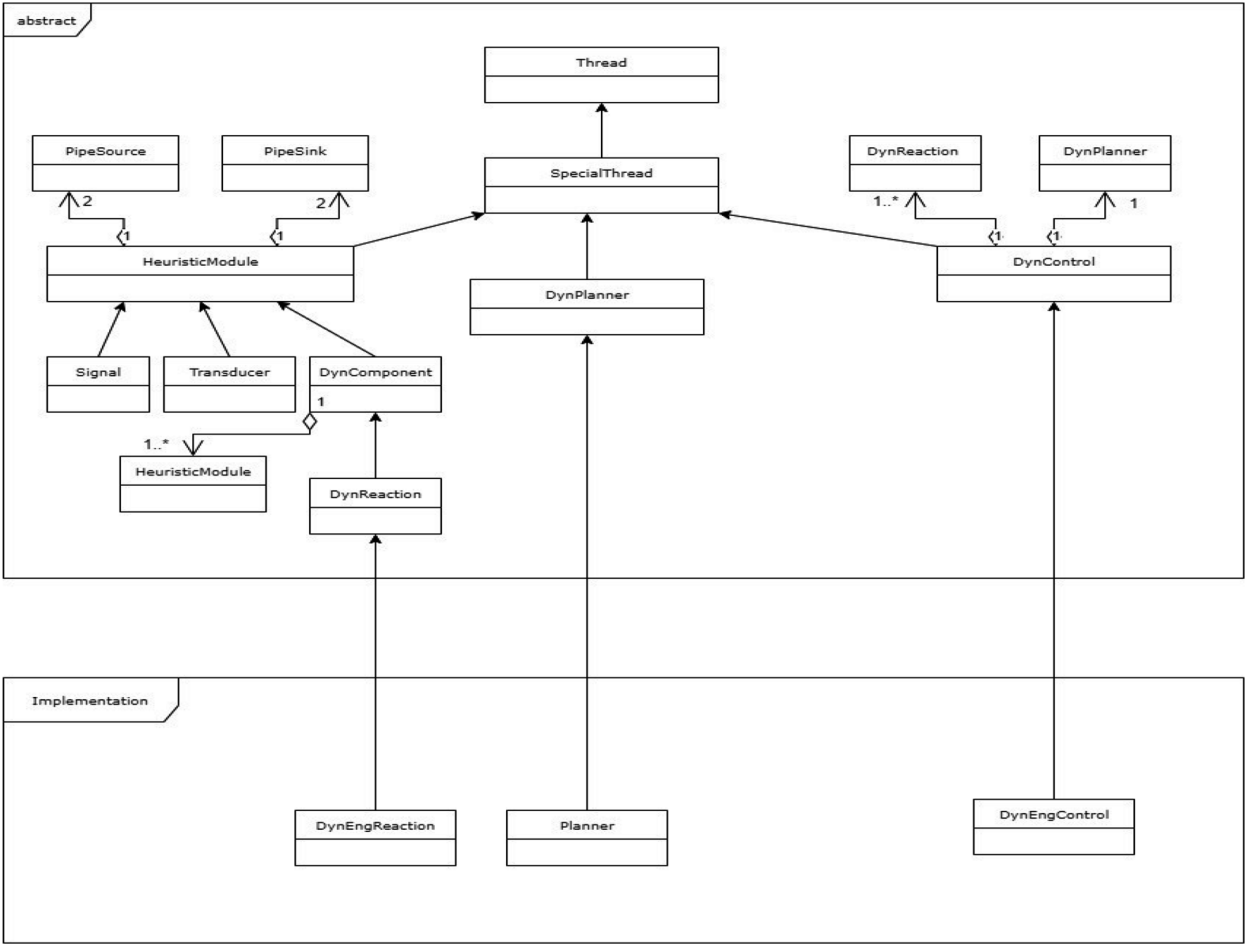
Class diagram of the framework. Source: Own elaboration.

The class diagram is shown in Fig. 5.

**Figure 6:**
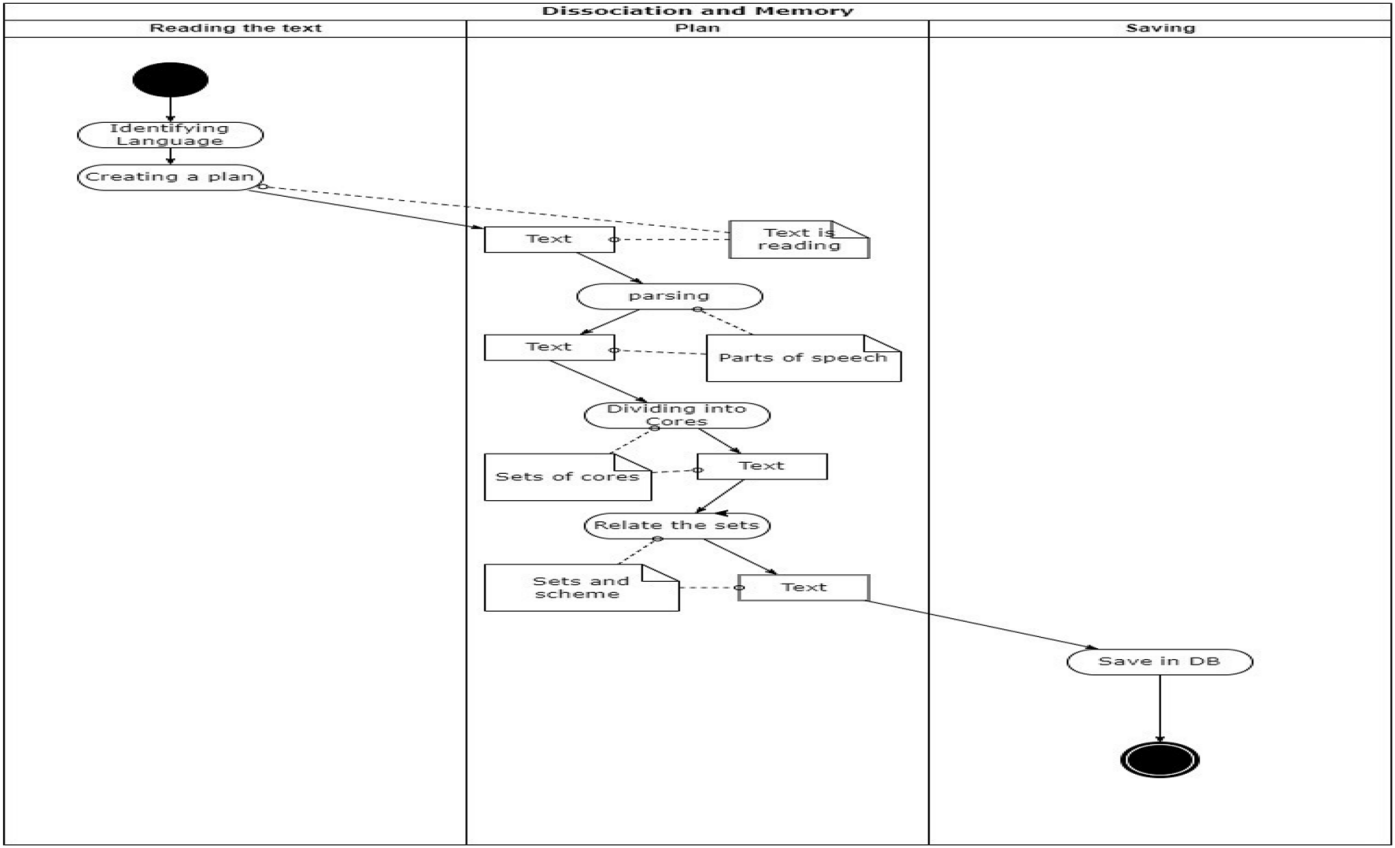
Activities diagram of the framework. Source: Own elaboration.

And the activities diagram is shown in Fig. 6, this last diagram is only for dissociating, because the recovery depends on the implementation which is shown in Section ”An approach”.

### Dissociation subsystem

The function of the dissociation subsystem is to split a text/sentence into special units. As previously mentioned in section *From sentences to clusters of words*, all languages share a common characteristic which is the identification of three basic clusters within a sentence: *Subject*(**S**), *Verb*(**V**), and *Object*(**O**). They can occur within a sentence in a different order depending on the language.

In this paper, the expression *SOV-trio* or simply *SOV* will be used to represent the trio that models a sentence or a text. Given that *Subject* and *Object* have similarities both will be treated as (**S**). Additionally, each of the components of a *SOV* will be named a *core*.

The cores may contain one or more words from the sentence. For example, it is possible to have a verb followed by another verb in the same core, as in the following sentence: *”Fred quiere ir a Hong Kong y visitar sitios turísticos”* the two verbs (”quiere ir”) constitute a *V* core. Once a *SOV* is generated, this is dispatched to the memory subsystem.

**Strategies to generate SOVs**

**Figure 7:**
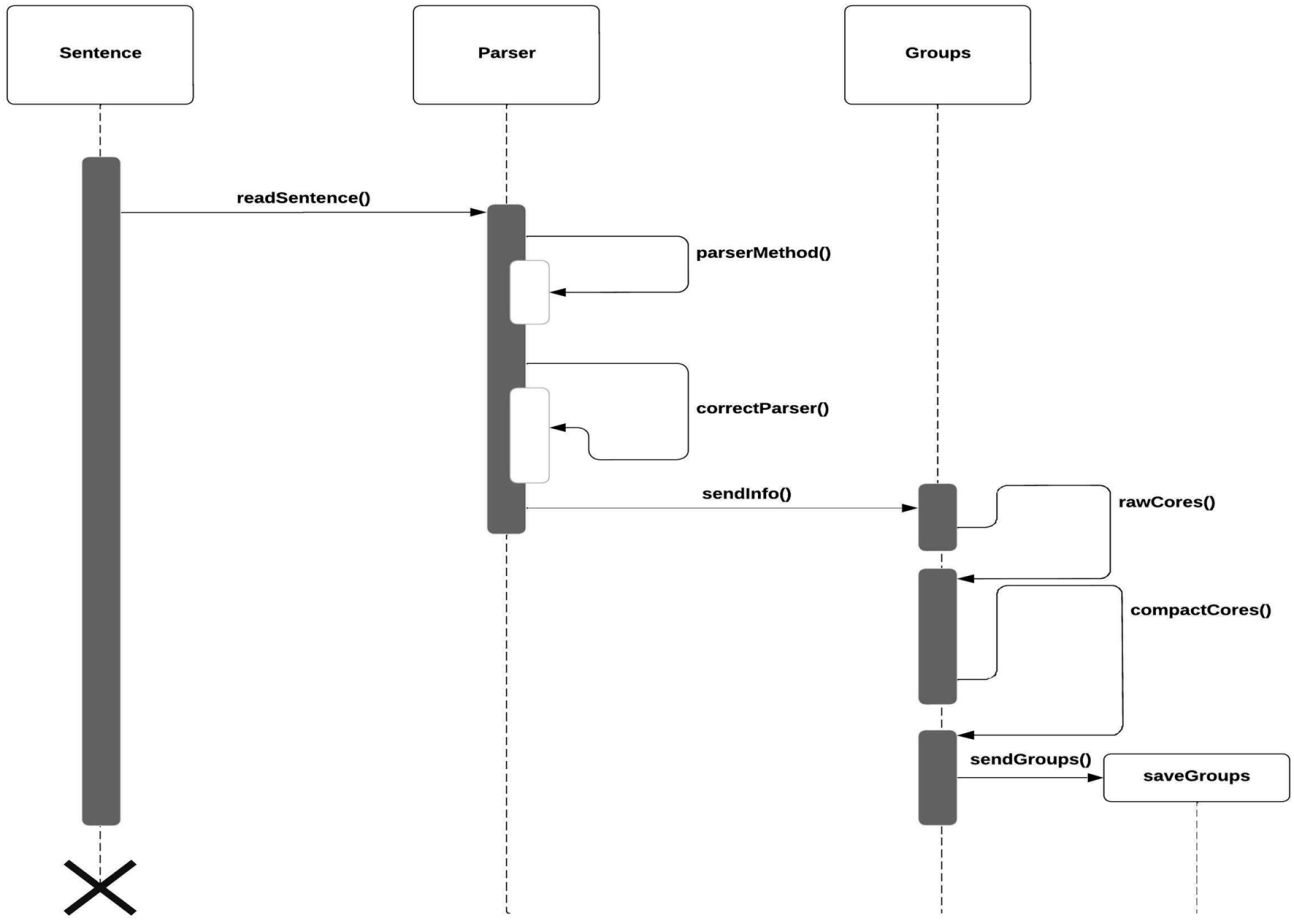
A generic UML sequence diagram for the *dissociation process*. Source: Own elaboration.

As explained in section *Object-action dissociation/integration*, there is a consensus about the dissociation between actions (verbs) and objects (nouns) inside the human mind. However,^32^ emphasizes the existence of problems by establishing the grammar category that can generate confusion between verbs and nouns, this also can happen in the process of dissociation in this subsystem. To dissociate the sentences correctly, the subsystem should implement modules such as:

- *Syntactic Analysis (Parsing)*. An ordinary parser generates a syntax tree from which the *SOVs* can be rapidly built. Although this strategy is good, it does not avoid that the syntax tree generated may require the involvement of some other heuristic processes to “refine” the creation of the cores, for instance, in cases of slang interpretation as is shown in Fig. 7, **Parser module** of, routine **parserMethod()**.
- *Dictionaries and conjugators*. Sometimes, parsers can produce an incorrect word classification, especially when the parser has not well-trained in a particular language, in such case it is necessary to perform an analysis and debugging process over these words. For this purpose, software like dictionaries and conjugators modules could be useful to validate the category as is shown in Fig. 7, **Parser module**, routine **correctParser()**.
- *Grouping of elements*. The dissociation in cores requires identifying elements like *determinants*, *adverbs*, *prepositions*, *conjunctions*, etc., in such a way that they will be inserted in the adequate core. This process should be customized for each language as is shown in Fig. 7, **Groups module**.

To summarize, some procedures, syntactic-semantic strategies, and heuristics should be implemented to help in building the *S/O/V cores* correctly.

### Memory subsystem

An important function of the memory system is to store the information generated by the dissociation subsystem. Hence, it is mandatory to build a structure that guarantees order and efficiency. Therefore, the memory system should contain a repository to save the *SOVs* generated by each text read interrelated between them. This storage should maintain these cores in such a way that can be retrieved in the exact order as they were read. According to these principles, the implementation should comply with the following conditions:

- **SQL-database**. The type of database towards has been addressed in this research is the SQL-database because it is the most used to store information. The main idea is to save the texts in such a way that their elements will be organized in groups or clusters representing sets that, joining them, can reproduce the source without losing their meaning.
- **Repository based on queries of cores**. Firstly, a repository based on query means that uses SQL technology to save and recover information. Secondly, the queries can be attended by modules that recover cores, compare against the queries, determine similarity, and create sentences, paragraphs, and full texts as of the cores chosen.
- **Meta-engine**. Each implementation should program a meta-engine that works over the database in a superior layer that the database engine, this should be equipped with the algebraic operation explained in subsection *Algebraic environment* and mappings between groups to integrate them and build a part or whole original text.

**Dynamic structure**

**Figure 8:**
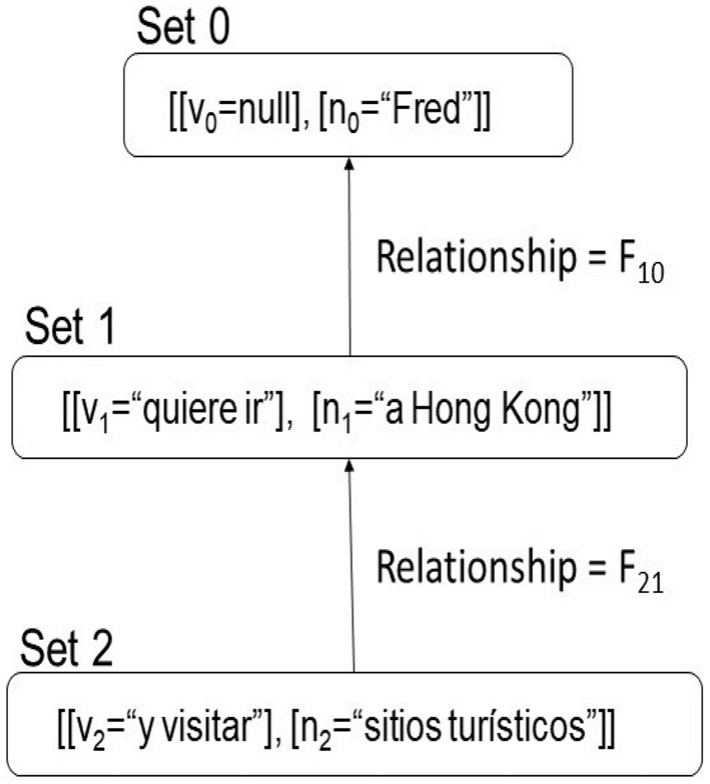
Relationship between groups generated by the sentence: ”Fred quiere ir a Hong Kong y visitar sitios turísticos”. Source: Own elaboration.

The dissociation in *SOVs* and the mapping create sets distributed and connected in terms of their original semantic content. Figure 8 shows a scheme that illustrates the relationship between the sets of *SOVs* (*Abelian group*).

The nodes will be related with adequate functions (mapping) to guarantee that the recovery of the part, or the whole, of a sentence/text will be executed correctly as will be explained later.

### Recovery subsystem

The purpose of this subsystem is to generate, in a dynamic way, a sentence/text part or entirely. This subsystem is closely interrelated to the *dynamic structure* because this subsystem is composed of the functions that connect the nodes.

**The Engine**

**Figure 9:**
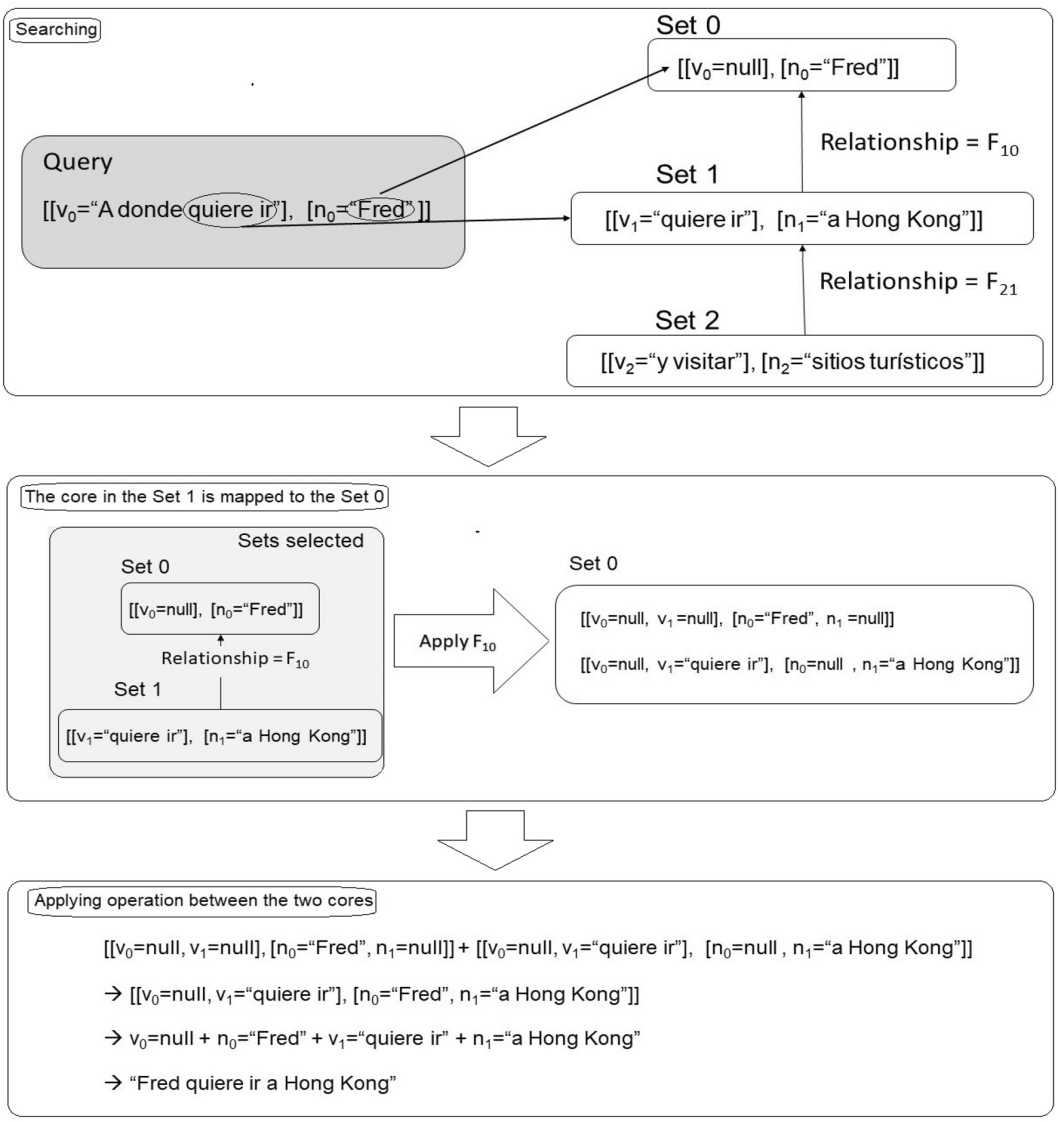
Creating a sentence from a query. Source: Own elaboration.

The queries are expected in natural language and it would transform into a set of *SOVs*. The key is to compare *SOVs* for finding the closest results. The strategies to match the *SOVs*.can be wide. An example could be to establish matches of *SOVs* that contain elements that could respond contextually to the query as in Fig. 9. The degree of coincidence will be the measure.

This strategy could recover sentences that do not answer the query completely, hence, it would be important to implement another stage. For instance, that compares sentences in a logical context. This can be carried out by converting the query and the text recovered into small text-theories that can be matched logically.

## An approach

This section has a summary of a prototype designed as a **layered framework** that could be used for any language characterized as S-V-O (Spanish, English, etc.) The most relevant layers of the dissociation processes are the following:

1. **Identifying the language**. This first layer has been designed to identify the language of the text and divide it into sentences, and finally, their results will send to the next layer one at a time.
2. **Planning**. This second layer chooses the modules required to dissociate the sentences based on the language recognized. This layer makes flexible the framework because it allows changing the rules of dissociation depending on the language to be processed.
3. **Reaction layer**. This layer is related to the strategies to generate *SOVs* which were described in the paragraph *Strategies to generate SOVs*. Figure 7 is shown the execution of two modules in a pipeline way, but new modules could be included to improve the results, this will depend on the implementation. The name of this layer is due to the modules chosen by the *plan layer* being triggered dynamically and executed like a chain reaction in a pipeline.

In this implementation, the modules created in the reaction layer dealt with sentences in the Spanish language (S-V-O language) and were organized in three linear phases following the guidelines described in the paragraph *Strategies to generate SOVs* and shown in Fig. 7. In the first phase, each sentence is processed by a linguistic tool, commanded by the VISL parser^51^ *reaction layer*, in this stage, it, also, corrects possible inconsistencies generated by the parser as the wrong classification of the words, e.g., some words classified as nouns or vice versa. The information produced by the parser is significant, therefore, it is discriminated, and sent to the next module in the pipeline. The second phase receives the information and classification and applies heuristics for generating, initially, *raw-clusters*, then refined by another heuristic, and finally to produce the set $O_v$. Lastly, in the third phase, the set $O_v$ is saved in a standard database (SQL-style). The heuristics applied in this approach are not extendable to other languages. However, currently, they are being tested in the English Language, also S-V-O language, to measure their effectiveness in it. Each sentence is organized in Abelian groups hierarchically organized with a binary operation capable of building phrases (see Fig. 3). The Abelian groups obey the specifications done in section *Algebraic environment*.

Table 2 shows the classification established heuristically for the Spanish language cores in this approach. This process involves a loop where neighboring words that comply with certain conditions are packed into a single class named: **nominal core (S)**, **determinant**, and **verbal core (V)**. A *determinant* is used to interrelate Abelian groups as in Fig. 8 in the paragraph *Dynamic structure*. It is important note that the punctuation signs are useful to create these categories, some are part of the **determinants** and other are par of the **verbal cores** or **nominal cores**, for example, in Fig. 8 the **nominal core** $n_6$ in $G_2$ include a comma: ”, la Paz y la justicia”, similarly, the **verbal cores** $v_5$ in $G_3$: ”, recibía”.

**Table 2:** Final categorization scheme.

**Category**	**Description**
*p, J*	*Determinants*
*v, V*	*Verbal core*
*n*	* Noun core*

In this approach, there are two types of determinants *p-det* and a *J-det*; both interrelate the sets with functions, but the Abelian group pointed by a *J-det* is considered optional in the rebuild of the sentence. All of these properties were established empirically.

**Figure 10:**
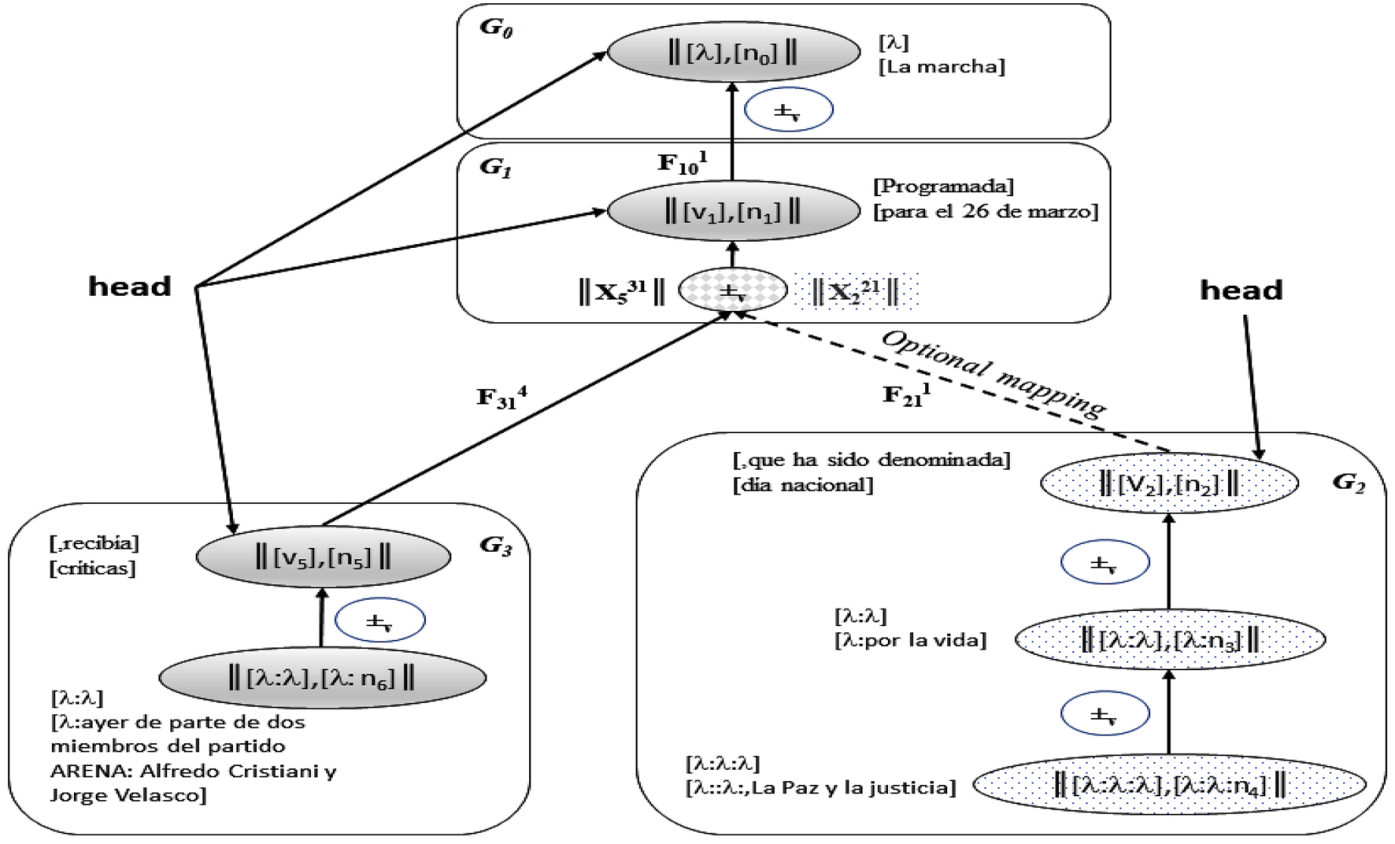
Scheme to recover sentences. Source: Extracted from^52^.

The restoring process is not the reverse operation exactly, else it is a complex process that executes tasks from the repository trying to preserve syntax and the original semantics. This purpose is successful due to the properties of the Abelian groups (see^52^) and the hierarchy of sets created by the determinants in the dissociation process. The process is shown in Fig. 10

The sets are operated and mapped in a domino way from the core where the matching occurs up until the root of the hierarchy. For example, in Fig. 10, if the core matched is in $G_3$ and corresponds to ”$n_2$ = críticas” then the recovered sentence will be: “*La marcha programada, para el próximo 26 de marzo, recibía críticas*”.

## Proposal comparison

Currently, generating sentences and small texts is a task very significant in several fields of Computer Science. The approach named **Rhetorical Structure Theory (RST)**^53^ is one of the first proposals created to divide the discourse and has been the inspiration for **Natural Language Generation** (**NLG**) schemes.

The strategy used in *RST* to divide the texts into cores and organize them hierarchically (nucleus and satellite) requires training of neural networks^54,55^. The relevance of the framework presented in this document is that it does not need training.

With respect to the generation of sentences, the implementations and approaches are very exigent. According to^56^, they should carry out several complicated tasks as below:

1. Determining the information relevant. This part is associated with the context and scope, basically related to the searching^57–60^.
2. Determining the order as the words should appear. Some approaches try to resolve this part from texts by collecting, recovering, and organizing sentences inside them^61,62^
3. Determining: how should be the information aggregated? This stage is considered very difficult because the information can be provided by several sources or it is not the correct response to any query. Some works use the context to resolve discrepancies or the domain to explore the sources^63,64^.
4. Determining the right words and phrases (verbs and nouns). This part contains two stages but they will be joined in one because can be carried out jointly. In this part the sentence is organized in one of the following structures: SVO, SOV, VSS, VOS, also, it is analyzed the verb times^65^.
5. Combining words and phrases to generate well-formed sentences. This phase builds the sentences, sometimes through templates, or grammar-based techniques, among others^66,67^.

In the framework exposed in this document, the first three steps are part of the recovery system in this framework, specifically, corresponding to the engine searching. the last two steps can be resolved by responding to the queries and executing the algebra of the groups and mapping between them which are tasks easy and efficient. All of these show a framework simple to implement.

## Conclusions and future works

The high demand for information has caused an increasingly important in the automation of processes such as decision-making, pattern recognition, and interaction human-machine, among others. Several of these processes require the use of the text, either to understand queries, generate reports, or answer in natural language, hence, building applications with these functions takes a greater relevance. This paper presents an architecture for dissociating the text/sentences, saving it in a SQL database, and recovering it without loss of meaning. This is highly productive in process automation because the textual information is converted from unstructured to structured format and the queries and other processes in natural language can be more efficient.

The suggested system has been inspired and based on processes verified by scientists related to the dissociation of the information inside the human brain, memory models in the Neuroscience field, and the structure of the languages in Linguistic and Psycholinguistic disciplines. The proposed framework divides a sentence/text into clusters like the brain dissociates the speech into nominal and verbal categories. The scheme will divide the text/sentence into sets of cores named *nominal cores* and *verbal cores*, and implement an algebraic operation that can be used to generate new sentences that keep the original meaning without loosing the structure of the language. This proposal was applied by the approach studied in the last section successfully.

The explored implementation resolved a great part of the challenges described in the paper by implementing a framework with abstract modules that can be custom implemented, for instance, the processes described in the architecture, the generic abstract modules for different languages, and the recovery modules, among others.

Additionally, the implementation creates a solution for the Spanish language by using heuristics for both dissociation and recovery processes. The application suggests interrelating the algebraic sets by employing functions to recover the whole or part of the textual information by maintaining the meaning. The approach shows that for the Spanish language is possible to have an implementation. In^68^ is exposed several proposals of NLG.

A system has been proposed for converting unstructured textual information to be computationally managed structured information. This proposal has been tested in an approach for the Spanish language successfully. Future works will be addressed to implement this framework for other languages and to generate applications for these approaches.

## Data Availability

The current document has been focused on the discussion about a framework able to compose strategies to divide sentences and texts into cores to save them in a SQL-databases. Under this context the data used to verify the effectiveness belong to other work where the purpose was to study heuristics for dividing the sentences into these clusters, these data do not form part of the current research. Therefore, all data generated or analyzed during this study are included in this published article.
